# Role of Iron Chelation and Protease Inhibition of Natural Products on COVID-19 Infection

**DOI:** 10.3390/jcm10112306

**Published:** 2021-05-25

**Authors:** Giuseppe Carota, Simone Ronsisvalle, Federica Panarello, Daniele Tibullo, Anna Nicolosi, Giovanni Li Volti

**Affiliations:** 1 Section of Biochemistry, Department of Biomedical and Biotechnological Sciences, University of Catania, 95123 Catania, Italy; giuseppe-carota@outlook.it (G.C.); d.tibullo@unict.it (D.T.); 2 Department of Drug Sciences, University of Catania, 95123 Catania, Italy; s.ronsisvalle@unict.it (S.R.); panafede11@gmail.com (F.P.); 3 Hospital Pharmacy Unit, Ospedale Cannizzaro, 95125 Catania, Italy; annanicolosi@hotmail.com

**Keywords:** SARS-Cov-2, COVID-19, iron chelation, protease inhibition, natural products

## Abstract

Although the epidemic caused by SARS-CoV-2 callings for international attention to develop new effective therapeutics, no specific protocol is yet available, leaving patients to rely on general and supportive therapies. A range of respiratory diseases, including pulmonary fibrosis, have been associated with higher iron levels that may promote the course of viral infection. Recent studies have demonstrated that some natural components could act as the first barrier against viral injury by affecting iron metabolism. Moreover, a few recent studies have proposed the combination of protease inhibitors for therapeutic use against SARS-CoV-2 infection, highlighting the role of viral protease in virus infectivity. In this regard, this review focuses on the analysis, through literature and docking studies, of a number of natural products able to counteract SARS-CoV-2 infection, acting both as iron chelators and protease inhibitors.

## 1. Introduction

Coronaviruses, named for the crown-like spikes on their surface, are a family consisting of enveloped single-stranded and positive-strand RNA viruses, possessing a helical nucleocapsid. They are known to cause acute and chronic respiratory and central nervous system diseases in animals and humans [1]. Severe acute respiratory syndrome coronavirus 2 (SARS-CoV-2) primarily affects the tissues expressing angiotensin-converting enzyme 2 (ACE2) receptor, including the lungs, heart, kidney and endothelium, leading to systemic manifestations [2]. Furthermore, the role of Neurolipin-1, abundantly expressed in the respiratory and olfactory epithelium, has been recently investigated as a significant enhancer of SARS-CoV-2 infectivity by promoting the interaction of the virus with ACE2 receptor [3]. The ongoing epidemic outbreak caused by the coronavirus disease 2019 (COVID-19) calls for international attention to develop effective therapeutics including selective vaccines. Nevertheless, no specific therapeutic is yet available, leaving patients to rely on general and supportive therapies, such as oxygen supply, glucocorticoid, and human serum albumin [4]. Although some therapies, such as antiviral drugs, Chloroquine, and recombinant monoclonal antibodies, are showing a promising efficacy, additional therapeutic options should be explored and new therapeutic targets should be considered when taking into account the increasing number of SARS-CoV-2 cases [5]. Among factors capable of affecting viral infections, iron plays a critical role and represents a double-edged sword that acts both on favoring viral progression and exacerbating inflammatory processes; on the other hand, the inhibition of proteases, crucial to impede the virus-host cell fusion, could be highlighted by exploiting the activity of unconventional drugs, such as natural products.

Dysregulated iron homeostasis is one of the potential causes of diffuse endothelial inflammation with systemic involvement, resulting in oxidative stress and inflammatory response [6]. A range of respiratory diseases, including acute respiratory distress syndrome (ARDS) and pulmonary fibrosis, have been associated with higher iron levels that may promote the course of viral infection [7,8,9]. Iron dependence on viral replication and the modulation of host iron metabolism exerted by virus infection highlight the importance of cellular iron homeostasis in the viral life cycle and lead to the development of iron chelation strategies in treating viral infections. Currently, few iron chelators have been approved by the U.S. Food and Drug Administration for clinical use, such as Deferoxamine and Deferasirox. With a strong and selective affinity with iron ions, these drugs can bind free iron and remove it from iron-storing proteins [10,11]. Recent studies have demonstrated that some natural components of the human innate immunity could act as a first barrier against viral injury and, in this regard, increasing interest has been shown in the possible preventive role of lactoferrin as adjunct treatment [12,13]. Lactoferrin is a glycoprotein of human secretion, belonging to a non-specific defensive system, known to play a pivotal role against viral infections and able to regulate iron metabolism. The main capability of Lactoferrin is to reversibly chelate two Fe^3+^ per molecule with high affinity, binding iron until pH values of 3.0 [14]. Through sequestering free iron and restoring iron homeostasis, Lactoferrin reduces oxidative stress and inflammation, principally associated with the cytokines storm and COVID-19 pathology [15,16,17].

Proteases are the enzymes involved in proteolysis, a protein catabolism by hydrolysis of peptide bonds. Proteolytic processes are necessary for normal physiological functions in the body, such as digestion, angiogenesis, and bone remodeling [18]. In enveloped viruses, post-translational proteolytic activation is a crucial step for the fusion with the host and thus for the infectivity of the virus. Both membrane receptors and proteolytic activation are indispensable for effective virus spread in the infected host, determining the level of the pathogenicity [19]. Proteases have been identified in a wide range of viruses, without any correlation to the envelope presence: cysteine proteases are present in adenoviruses, while the family of aspartyl proteases has been found in human immunodeficiency virus of type 1 (HIV1). Furthermore, the proteases present in various viruses specifically belong to the family of serine protease, as in hepatitis C virus (HCV), herpesvirus, and in SARS-CoV-2 [20]. Several protease inhibitors have been considered as drugs of choice to counteract the infection of viruses whose entry into the host cell is strictly related to proteases activity, including SARS-CoV-2 [21,22,23]. Few recent studies have proposed the combination of existing drugs involving anti-HIV drugs (lopinavir/ritonavir, lamivudine, tenofovir) for therapeutic use against SARS-CoV-2 infection: the consequent recognition of proteases as a new attractive target led to a deep investigation of alternative compounds able to target viral serine protease [24,25]. With this purpose, a recent in-silico study reported the potential of a few phytoconstituents (including glycyrrhizin, tryptanthrine, rhein, and berberin) to target the main protease involved in COVID-19 activity; the obtained results showed a high degree of interaction with the viral protease accompanied by low binding energy, thus leading to favorable drug-like properties exerted by natural compounds [26].

Starting from these assumptions, it is possible to suggest that a number of natural products, whose effect as iron chelators and/or viral protease inhibitors has been proven (Table 1), could show the capability to control and reduce the inflammatory condition and the relative oxidative stress raised after SARS-CoV-2 infection, furthermore acting by preventing virus replication.

## 2. Iron and SARS-CoV-2

Iron is an essential element that plays a pivotal role in many cellular processes that are necessary for life, including oxygen transportation, oxygen sensing, electron transfer, energy metabolism, and DNA synthesis [27]. Iron is required for viral replication and other processes including ATP generation, cell survival, ferroptosis, and DNA/RNA synthesis and repair [28]. Low intracellular iron levels are sufficient to support coronavirus replication, whereas iron deficiency interferes with viral transcription, translation, assembly, and exocytosis [29]. The main site of iron storage (in its ferric state) is Ferritin, that can carry up to 4500 iron molecules in its core [30,31]. Systemic inflammations are generally associated with increased serum ferritin levels: indeed, during strong inflammation state, cytokines stimulate ferritin and the hepcidin synthesis, the main regulator of the tissue iron store [32]. Regarding this, a high level of ferritin has been reported in patients with COVID-19 disease [33,34,35]: on one hand, SARS-CoV-2 attacks one of the beta chains of the hemoglobin, which leads to the dissociation of iron from heme and the consequent increased free iron and ferritin levels in the body [11,34,36,37,38]; on the other hand, one of the causes has been associated with the inflammation induced by COVID-19 infection, with a remarkable overexpression of IL-6, IL-1β, and IFN-γ, leading to the increase of the hepcidin level [6,39,40]. Hepcidin, as key iron regulatory hormone, sequesters iron in the enterocytes and macrophages, enhancing intracellular levels of ferritin and preventing iron efflux from store cells through the inhibition of the iron-exporting protein ferroportin [41]. Ehsani recently supposed a similarity between the hepcidin protein and the distant amino acid sequence of the SARS-CoV-2 spike glycoprotein cytoplasmic tail, highlighting the potential route of investigation of factors that interplay between cytokine-mediated inflammatory processes, respiratory infections, and systemic iron regulation (Table 2) [42].

## 3. Protease Inhibition and SARS-CoV-2

As reported in the literature, infections by SARS coronaviruses are dependent not only on the host ACE2 receptor but also on the priming of the virus’s spike (S) protein by the Transmembrane Serine Protease 2 (TMPRSS2). The cleavage of the S protein is the necessary step that leads to the membrane fusion of virus and host and the consequent cell entry [43,44]. Although further clinical data are needed to prove it, several studies suggested the use of protease inhibitors for the prevention of COVID-19, recommending the administration of antiretroviral drugs, such as the lopinavir/ritonavir combination for the initial clinical management of patients with SARS-CoV-2 infection [25,45,46]. In addition, it has been shown that nafamostat mesylate and camostat mesylate, both potent inhibitors of TMPRSS2, can be used to block SARS-CoV-2 cell entry as promising prophylactic options for the clinical manifestation of COVID-19 infection in critically ill patients, especially in cases with possible coagulopathies [25,43,47,48,49,50]. With the purpose to focus on SARS-CoV-2 protease for the development of new therapeutical strategies, a recent computational study highlighted the possibility to discover and identify new lead compounds able to target main protease (M^pro^) of SARS-CoV-2 that represent a key coronaviruses enzyme crucial for replication and transcription [51].

## 4. Materials and Methods

The docking studies were performed according to the protocol used in a paper previously published [52]. All compounds were built and their energy minimized with “Flare preparation ligand” [52]. The protease coordinates were downloaded from the protein databank with the code 6Y2E that possess a resolution of 1.75 angstrom. According to the literature, the binding site was identified in a specific region named TAEDMLN. The catalytic domain has been located between residue 41 and residue 145, specifically from amino acids 45 to 51, right charges were calculated with “Flare protein preparation”. Docking calculation were performed with Flare with the “Accurate but Slow” setting that provides the dG score which provides an accurate estimation of the free energy of protein-ligand binding. The best docking poses were refined by ligand-protein complex energy minimization using Flare. Finally, to improve the stability of each complex, short (5ns of production) molecular dynamic runs were performed at a constant temperature followed by a quick minimization of all the atoms involved in the binding site.

## 5. α-Lipoic Acid

**Figure 1:**
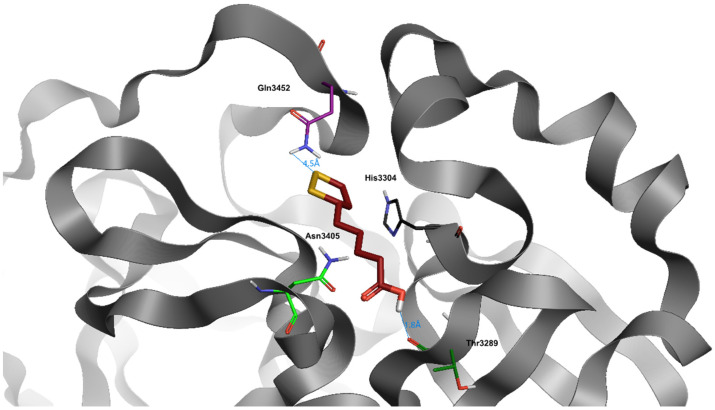
Docking position of α-lipoic acid (dark red) in the main protease of SARS-CoV-2 virus binding site. Water and hydrogen were omitted for clarity.

Alpha-lipoic acid (ALA), also known as 1,2-dithiolane-3-pentanoic acid and thioctic acid, is a natural substance existing in almost all types of prokaryotic and eukaryotic cells (Figure 1). The ability as antioxidant and metal chelator of ALA allows the reduction of the oxidized forms of several antioxidant agents, such as glutathione (GSH) and vitamins E and C [53]. The efficacy of ALA as iron chelator has been demonstrated in a combined treatment with ferric ammonium citrate (FAC), simulating an iron overload condition both in vitro and in vivo. Administration of ALA in mesenchymal stem cells leads to a decrease of reactive oxygen species (ROS) levels and a restoring of mitochondrial membrane potential and integrity, following the treatment with FAC. Augmented levels of GSH have been associated with the direct antioxidant effect of ALA, leading to enhanced antioxidant defenses for the cells. Consequently, through the increase of intracellular GSH content, ALA prevents the nuclear factor erythroid 2–related factor 2 (NRF2) pathway activation, leading to the reduction of heme oxygenase 1 (HO-1) expression. The same result has been confirmed in an in vivo model of zebrafish, with significant reduction of heme oxygenase 1b (HMOX1b), mitochondrial superoxide dismutase (mtSOD), and ferroportin 1 (FPN1) expression after treatment with ALA in the presence of an iron overload [54,55]. Following different in vivo experiments, Zhao et al. demonstrated the capacity of ALA as iron chelator to prevent the light-induced retinal degeneration in a mouse model of AMD (age-related macular degeneration) through systemic administrations [56]. Abnormally high levels of iron in the brain have been demonstrated in a number of neurodegenerative disorders, such as Alzheimer’s disease and Parkinson’s disease (PD). The neuroprotective effect of ALA was tested in a PD model induced by 6-hydroxydopamine (6-OHDA), showing significant reduction of ROS and an improvement of iron metabolism levels, confirming the therapeutic potential of ALA for the treatment of neurodegenerative diseases associated with iron metabolism dysfunction and oxidative stress [57].

Alpha-lipoic acid shows an excellent dG (−8006) (Table 3). Naturally, the small steric footprint of this compound allows it to enter into the bonding site with ease.

It also establishes stable hydrogen bridges throughout the dynamics time with Thr3289, through the carboxylic group of the molecule, and with Gln3452 via an NH-S bond.

## 6. Quercetin

**Figure 2:**
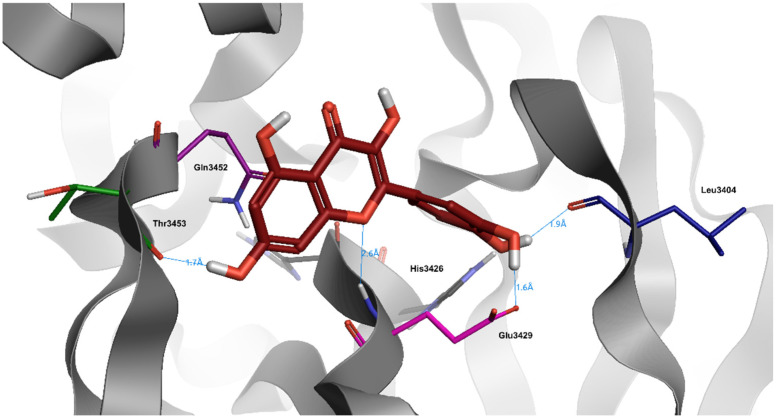
Docking position of quercetin (dark red) in the main protease of SARS-CoV-2 virus binding site. Water and hydrogen were omitted for clarity.

Quercetin is the most abundant dietary flavonoid, especially enriched in onions, cranberry, blueberry, tea, and apples (Figure 2) [58]. Many polyphenol compounds, including quercetin, are potent iron chelators. In common with most polyphenols, quercetin is found almost exclusively in foods as glycoside conjugates but can be converted rapidly into the aglycone in the intestinal lumen via the actions of glycosidases [59]. Quercetin is able to exert a significant decrease of non-heme iron absorption in duodenum through different mechanisms: increasing iron uptake and retention by the duodenal mucosa; reducing tissue iron pool and expression of non-heme iron transporters in enterocytes; increasing hepcidin expression, leading to iron depletion [60]. Oral administration of quercetin, in an in vivo model of rat, led to the downregulation of divalent metal transporter-1 (DMT1) and FPN mRNA levels; conversely, following the same experiment in an in vitro model with Caco-2 cells, quercetin did not affect DMT1 and FPN mRNA or protein expression [61]. It has been shown that treatment with quercetin in an in vivo model of iron overload did not demonstrate significant differences compared to treatment with Deferoxamine, leading to the reduction of serum and tissue iron and enhancing the inflammatory condition by reducing IL-6 and increasing IL-10 expression [62]. A wide range of studies reported the potent ability of quercetin as protease inhibitor when used against virus infection: Bachmetov et al. demonstrated a direct inhibitory effect of quercetin on HCV NS3 serine protease catalytic activity, while Yao et al. showed that quercetin potently inhibited Enterovirus 71 (EV71) 3C-protease activity, blocking EV71 replication [63,64]. The same anti-replication activity has been investigated in computational studies on Middle East respiratory syndrome-coronavirus (MERS-CoV) and SARS-CoV, where results proved a strong interaction between quercetin and the catalytic site of viral 3C-like protease [65,66,67]. It has also been reported that co-administration of quercetin, as a promising inhibitor of crucial viral enzymes (reverse transcriptase, integrase and protease), and Vitamin C could be suggested for prophylaxis in a high-risk population and for the treatment of COVID-19 patients as support therapy: the synergistic antiviral action could be due to the overlap of multiple properties of the different compounds, such as antioxidant and immunomodulatory properties, and to the capability of ascorbate to recycle quercetin, increasing its efficacy [68].

Quercetin has a very particular structure with a high number of phenolic groups, and it is capable of generating hydrogen bonds with Glu3429, His3426, and Leu3404, also proven by its docking score (−8.0) (Table 3). The tri-hydroxy-chromium-4-onic portion is always water exposed throughout the dynamics, probably due to its extensive positive charge.

## 7. Caffeic Acid

**Figure 3:**
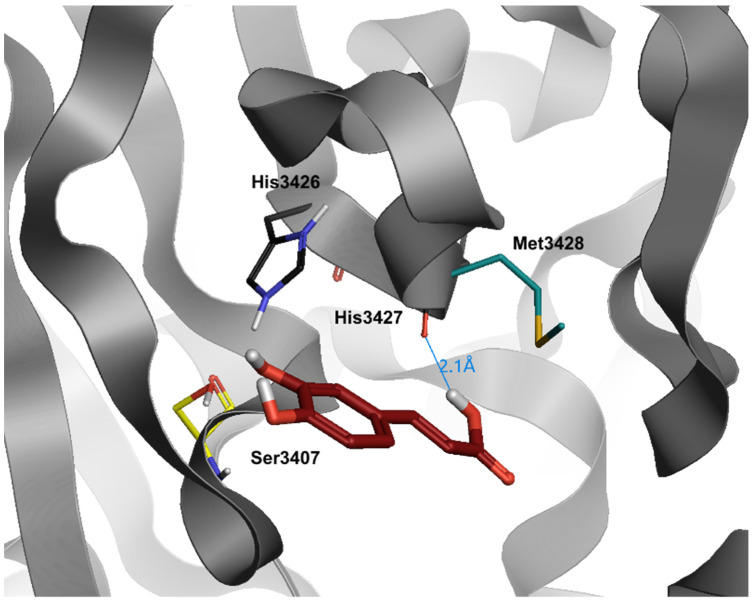
Docking position of caffeic acid (dark red) in the main protease of SARS-CoV-2 virus binding site. Water and hydrogen were omitted for clarity.

Caffeic acid (CA) is a phenolic compound produced by the secondary metabolism of plants and is the major hydroxycinnamic acid present in the human diet (Figure 3). Several studies have determined the ability of CA and its metabolites to bind metal ions, and iron in particular, and to affect the redox reactions mediated by these metals, such as Fenton reaction [69,70,71]. Moreover, antiviral activity of CA and related compounds with the caffeoyl moiety, such as rosmarinic acid, has been reported. Although the mechanism of action has not yet been determined, evidence suggests that iron chelators may target the extracellular attachment between the virion glycoprotein B and the heparan sulfate proteoglycans on the cell surface. The viruses most affected by the CA-iron complexes, that can utilize heparan sulfate proteoglycans for cellular attachment, are herpes simplex viruses (HSV1 and HVS2), influenza A and human immunodeficiency virus (HIV) [72]. It has been showed that the activity of CA on HIV is not related only to iron complexes formation: Wang et al. demonstrated that CA derivatives possess inhibitory activities towards HIV proteases, in addition to the known inhibitory activity on HIV integrase, suggesting the use of CA as lead compound to develop new potential anti-HIV drugs [73]. The inhibitory effect of CA phenethyl ester derivatives on viral protease have also been studied on HCV, where CA compounds showed the capability to decrease NS3 protease expression, leading to the reduction of virus replication [74]. Recent papers reported the noteworthy inhibition on the main SARS-CoV-2 protease M^pro^ exerted by CA derivatives: both studies showed that CA derivatives bind to the substrate-binding pocket of SARS-CoV-2 M^pro^ possessing more efficacy and binding energies than nelfinavir, an already claimed N3 protease inhibitor [75,76].

Partially similar to quercetin, but simplified in the structure, caffeic acid maintains the possibility to enter deepest in the bond pocket, but, as opposed to the previous molecule, it loses the ability to establish a high number of hydrogen bonds (dG = −6.3) (Table 3). In fact, the only hydrogen bond occurs with the carboxylic portion towards the His3427 in its portion of the backbone. In addition, the layout of caffeic acid, during dynamics simulations, is more changeable compared to other compounds.

## 8. Phytic Acid

**Figure 4:**
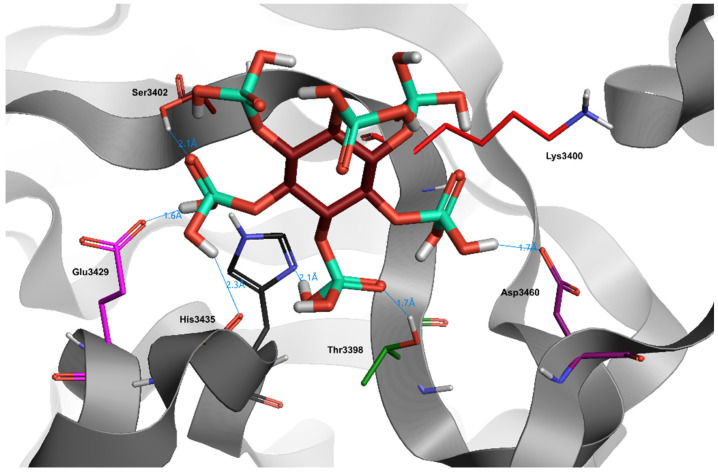
Docking position of phytic acid (dark red) in the main protease of SARS-CoV-2 virus binding site. Water and hydrogen were omitted for clarity.

Phytic acid (myo-inositol hexaphosphate, IP6) has been recognized as a potent antioxidant and inhibitor of iron-catalyzed hydroxyl radical formation under in vitro and in vivo conditions (Figure 4). IP6, a common content of cereals and legumes, has been generally considered as an antinutrient because of its ability to chelate divalent minerals and reduce their absorption and is also considered as beneficial because of the same property [77,78]. Phytic acid was shown to inhibit radical OH formation and decrease lipid peroxidation catalyzed by iron and ascorbic acid in human erythrocytes [79]. Xu et al. reported that IP6, in a cell model of Parkinson’s disease, protected dopaminergic neurons against 1-methyl-4-phenylpyridinium (MPP^+^) induced apoptosis, in the presence of an iron-excess condition [80]. IP6 was also tested to ameliorate the pulmonary inflammation and fibrosis raised after intratracheal instillation of asbestos in rats: since iron-dependent enzymes are necessary for collagen secretion, such as prolyl hydroxylase and lysine hydroxylase, IP6 showed the important ability, as iron chelator, to control the fibrosis raised during pulmonary toxicity. Furthermore, IP6 was suggested to limit lymphocyte functions that contribute to pulmonary fibrosis [81]. Other studies demonstrated the antioxidant activity of IP6 as iron chelator in the inhibition of lipid peroxidation. IP6 was capable of inhibiting linoleic acid autoxidation and Fe^2+^/ascorbate-induced peroxidation, as well as Fe^2+^/ascorbate-induced lipid peroxidation in Caco-2 cells [82]. Moreover, Miyamoto et al. demonstrated the strong iron ion-chelating ability both of IP6 and its hydrolysis products (IP2, IP3, IP4, and IP5), able to prevent iron ion-induced lipid peroxidation. Results showed that subproducts of IP6, containing three or more phosphate groups, maintained the ability to inhibit lipid peroxidation, although their effectiveness decreased with dephosphorylation [83].

Despite the numerous phosphate groups and the consequent high total binding capacity, the sterically complex structure of Phytic Acid impedes a facilitated insertion into the receptor pocket, proved by a low docking score (−1.5) (Table 3). This result is justified by the excessive water exposure of the molecule. In fact, although Phytic Acid is capable of forming hydrogen bonds with a single aminoacidic binding site (Glu3429) and with other external sites (His3435, Tyr3398, Asp3460, Ser3402), the obtained bonds result unstable, due to the interference given by the surrounding waters.

## 9. Curcumin

**Figure 5:**
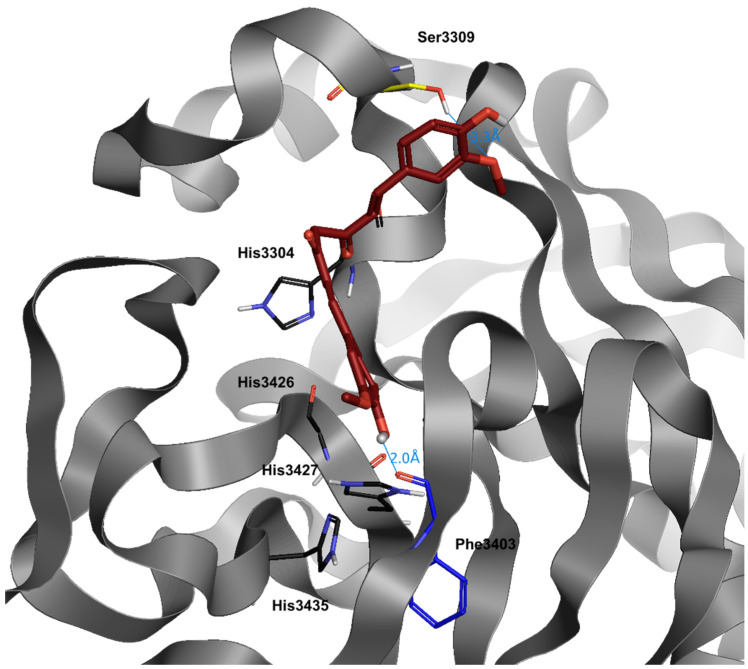
Docking position of curcumin (dark red) in the main protease of SARS-CoV-2 virus binding site. Water and hydrogen were omitted for clarity.

Curcumin (diferuloylmethane, or (E, E)-1,7-bis (4-hydroxy-3-methoxyphenyl)-1,6-heptadiene-3,5-dione) is a natural yellow colored product extracted from the Indian herb turmeric (Figure 5) [84]. A wide range of beneficial pharmacological effects has been proved for curcumin, including anti-inflammatory, antioxidant, antiviral, and antitumorigenic effects [85,86]. Curcumin has been reported to modulate proteins of iron metabolism in cells and in tissues, confirming the properties of an iron chelator. Because of its polyphenol structure, curcumin forms complexes with a number of different metal ions and especially with iron [87]. By inhibiting the Fenton reaction and other iron-catalyzed pathways of oxidative stress, curcumin is able to act as a chemopreventive agent, reducing oxidative injury to critical cellular targets, including DNA, lipids, and protein [88]. Jiao et al. demonstrated how curcumin affects the iron homeostasis, using an in vivo model of mice with low levels of body iron, describing different ways to cause iron depletion. Curcumin caused a dramatic reduction on hematological parameters of iron metabolism, such as hemoglobin, hematocrit, serum iron, and transferrin saturation. Moreover, curcumin positively affected the activity of iron regulatory proteins (IRPs); under iron-deficient conditions, these proteins are activated, leading to translational ferritin repression. Conversely, under iron overload conditions, IRPs are inactivated, thereby increasing the translation of ferritin mRNA [89]. In mice that had received the combination of high dietary iron and curcumin, IRPs activity was reported as significantly increased [90]. It has been reported that a high dose of curcumin can upregulate hepcidin and its regulators, such as bone morphogenic protein (BMP-6) Sekelsky Mothers Against DPP (SMAD) and transferrin receptor 2 (TfR2), in a mouse model of aplastic anemia with iron overload. The treatment with curcumin protected hematopoiesis from immune and iron overload-induced apoptosis, exerting an iron chelation effect in vivo more effective than Deferoxamine [91]. Since 1990s, numerous studies have focused on the antiviral properties of curcumin, investigating its activity as protease inhibitor and subsequently finding notable results on HIV protease inhibition and the collateral decrease of HIV replication [92,93,94]. Other works performed on flaviviruses (Dengue virus and Zika virus) found an allosteric inhibition of NS2B-NS3 proteases, exerted by curcumin by its binding to a cavity with no overlap with the active site, suggesting the use of curcumin as lead compound to design new small molecule allosteric inhibitors [95]. According to the study performed on the anti-SARS-CoV activity of a wide range of phytocompounds, Wen et al. demonstrated a significant inhibitory effect of curcumin on SARS-CoV 3CL protease activity, which is essential for virus replication, providing promising evidence for curcumin as a potential anti-SARS-CoV agent [96,97]. For this purpose, in recent years, several molecular docking studies have been performed that suggest curcumin would be effective at inhibiting SARS-CoV-2 replication through a range of ways, including the inhibition of the main viral protease [98,99,100,101]. Data obtained suggested that the chemical derivatives of curcumin could present a significant activity against COVID-19 disease by inhibiting the SARS CoV-2 main protease enzyme [99,101,102,103].

Curcumin has a great bonding capacity in the pocket of the viral protease: its high rotational capacity structure allows it to adapt to the receptor pocket in an optimal way, as demonstrated by its dG (−8.1) (Table 3). Curcumin’s best docking positions are achieved with Ser3309, due to the methoxylic moiety (in the apical portion of the protease) and with Phe3403, due to the opposite phenolic moiety.

**Table 1:** Effect of natural products on COVID-19 related diseases.

Natural Product	Effect	Reference
Lactoferrin	Reduction of oxidative stress and inflammation through iron chelation properties	[17]
Glycyrrhizin	Inhibition of SARS-CoV-2 protease M^pro^	[26]
Tryptanthrine	Inhibition of SARS-CoV-2 protease M^pro^	[26]
Rhein	Inhibition of SARS-CoV-2 protease M^pro^	[26]
Berberin	Inhibition of SARS-CoV-2 protease M^pro^	[26]
Quercetin	Inhibition of SARS-CoV-2 protease 3CL^pro^	[66]
Caffeic acid	Inhibition of SARS-CoV-2 protease M^pro^	[76]
Curcumin	Inhibition of SARS-CoV-2 protease 3CL^pro^	[97]

**Table 2:** Iron involvement in COVID-19 disease.

Type of Study	Iron Metabolism	Reference
Clinical	Anemic patients had increased levels of inflammation markers; Hyperferritinemia; Correlation between increased ferritin levels and cytokine mRNA over-expression	[33]
Meta-analysis	Elevated levels of serum ferritin have been found in non-survivors compared with survivors	[34]
Meta-analysis	The ferritin level was significantly increased in severe patients compared with non-severe patients	[35]
Clinical	High ferritin levels	[36]
Clinical	High ferritin levels	[37]
Meta-analysis	SARS-CoV-2 attacks one of the beta chains of the hemoglobin, causing dissociation of iron from the porphyrins and its release into the circulation	[38]
Clinical	Iron overload and Hepcidin overexpression	[40]
Computational	Similarity between hepcidin and the coronavirus spike glycoprotein	[42]

**Table 3:** Natural products and their structures with docking score (dG).

Name	Structure	dG
α-Lipoic Acid	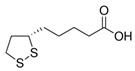	−8.0
Quercetin	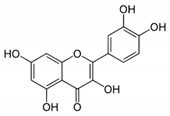	−8.0
Caffeic Acid	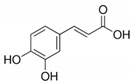	−6.3
Phytic Acid	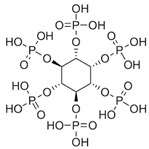	−1.5
Curcumin	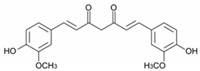	−8.1

## 10. Conclusions

During the COVID-19 disease, increased iron levels in the body generate reactive oxygen species which cause oxidative stress and damage to the lungs, leading to subsequent lung fibrosis and the decline in the lung function [7]. Evidence shows that iron overload increases viral replication, playing an important role in the severity of the infection [27]. Through their iron chelation effect, substances like Deferoxamine and Lactoferrin were shown to reduce iron availability in the serum and body tissue, preventing lung injury and fibrosis following COVID-19 infection [11,12]. In parallel, recent results nominate compounds with a potential as protease inhibition as new candidates to face SARS-CoV-2 infection [104,105]. In our review, we exposed and summarized the effects of some important natural products with an amply demonstrated activity as iron chelators and protease inhibitors, suggesting their involvement for new possible therapeutical strategies towards the pathological contexts related to COVID 19 infection.

## Data Availability

Not applicable.
